# Wrist Hypothermia Related to Continuous Work with a Computer Mouse: A Digital Infrared Imaging Pilot Study

**DOI:** 10.3390/ijerph120809265

**Published:** 2015-08-07

**Authors:** Jelena Reste, Tija Zvagule, Natalja Kurjane, Zanna Martinsone, Inese Martinsone, Anita Seile, Ivars Vanadzins

**Affiliations:** Institute for Occupational Safety and Environmental Health, Riga Stradins University, Dzirciema Street 16, Riga LV 1007, Latvia; E-Mails: tija.zvagule@rsu.lv (T.Z.); natalja.kurjane@rsu.lv (N.K.); zanna.martinsone@rsu.lv (Z.M.); inese.martinsone@rsu.lv (I.M.); anita.seile@rsu.lv (A.S.); ivars.vanadzins@rsu.lv (I.V.)

**Keywords:** computer mouse, cold hands, digital infrared imaging, ergonomics, hypothermia, sedentary work, wrist temperature

## Abstract

Computer work is characterized by sedentary static workload with low-intensity energy metabolism. The aim of our study was to evaluate the dynamics of skin surface temperature in the hand during prolonged computer mouse work under different ergonomic setups. Digital infrared imaging of the right forearm and wrist was performed during three hours of continuous computer work (measured at the start and every 15 minutes thereafter) in a laboratory with controlled ambient conditions. Four people participated in the study. Three different ergonomic computer mouse setups were tested on three different days (horizontal computer mouse without mouse pad; horizontal computer mouse with mouse pad and padded wrist support; vertical computer mouse without mouse pad). The study revealed a significantly strong negative correlation between the temperature of the dorsal surface of the wrist and time spent working with a computer mouse. Hand skin temperature decreased markedly after one hour of continuous computer mouse work. Vertical computer mouse work preserved more stable and higher temperatures of the wrist (>30 °C), while continuous use of a horizontal mouse for more than two hours caused an extremely low temperature (<28 °C) in distal parts of the hand. The preliminary observational findings indicate the significant effect of the duration and ergonomics of computer mouse work on the development of hand hypothermia.

## 1. Introduction

The modern world is unimaginable without computers and digital technologies. The number of computer users has progressively grown over the last three decades [1,2]. More and more people use computers for long hours at work as well as during their leisure time [1,2,3,4]. The duration of time spent physically inactive at a computer is frequently not felt by people in terms of physical discomfort [5,6,7,8,9,10,11,12]. Computer work is characterized by a sedentary, mostly static workload that results in low-intensity energy metabolism [1]. Emerging health risks from prolonged sitting and sedentary lifestyle are well known and include the development of obesity, cardiovascular disorders, diabetes, cancer, a decline in venous circulation in the lower extremities and pelvis, and impairment in bowel motility [1,13,14,15,16,17,18,19]. Prolonged time spent at a computer may also lead to musculoskeletal pain in the neck, shoulders, and lower back [20,21,22]. Work-related hand disorders like carpal tunnel syndrome and pain in the forearm and wrist may be found in computer users as well [1,22]. Many of these conditions develop due to an insufficient quantity of body movements and due to the permanent muscular contraction required for maintaining a stable posture for a prolonged time period [1,23,24]. Such inactivity results in the decline of microcirculation in the extremities and organs, impaired oxygenation of tissues, and disturbed elimination of metabolic waste products [1,25,26,27,28,29]. Muscle work requires a high amount of energy and produces heat as a side product [1,30]. It is important to note that most biochemical reactions in the human body need a normal body temperature for optimal action [31,32]. The disuse of muscles causes a lower level of energy metabolism and reduced production of heat [1,30,33,34]. It is also evident that sedentary work requires a higher ambient temperature for the comfort of employees, which has been endorsed in many countries by regulations for working environment [35,36,37,38]. 

Previous research mainly investigated microcirculatory conditions, muscular effects, and metabolic processes in computer work and a sedentary lifestyle, while thermal effects received less attention [1,20,21,22,25,26,27,39,40,41]. Some studies evaluated the changes in the surface temperature of hands in computer users [39,42,43]; however, the duration of the work evaluated was no longer than 15 minutes even though the usual amount of time spent at a computer is considerably longer. Moreover, our clinical observations of software developers indicated an appearance of “cold hands” after prolonged computer work despite a relatively high ambient temperature. At the same time there are a lot of authorized patents of inventions of heaters for hands for computer users and commercial interest in such devices is high [44,45,46]. Considering the low intensity of metabolism and impaired microcirculation during sedentary work and the lack of research on the dynamics of temperature changes in distal parts of the body during sedentary behavior and computer work, we hypothesize that different positions of the wrist through different computer mouse designs might influence hand temperature. In the current study, medical infrared digital imaging was chosen for skin surface temperature measurements. This is a method that allows a body surface temperature map to be made by detecting the infrared radiation coming from the body. It is a relatively simple, non-invasive, and precise method for the measurement of body surface temperature [47]. The aim of our study was to evaluate the dynamics of skin surface temperature in the hand during prolonged computer mouse work under different ergonomic setups.

## 2. Experimental Section 

### 2.1. Study Design and Setting

A descriptive observational study was conducted and designed to imitate routine sedentary work at a computer for a duration of three hours under controlled constant ambient environmental conditions. We assessed the dynamics of hand surface temperature changes during different computer mouse ergonomic setups.

### 2.2. Ethics 

The study was approved by the ethics committee of Riga Stradins University. Participation in the trial was voluntary. All study participants received clear verbal and written description of the aim and procedures of the study, as well as understandable instructions on their behavior (*i.e.*, a list of allowed and forbidden activities) during the study. Participants were allowed to opt out of the study at any time. Written informed consent was obtained from all participants.

### 2.3. Sample Characteristics

Four healthy young persons (two males and two females) were randomly selected from the office workers of four companies, where they regularly spend time working with a computer. The selection criteria for study participants were:

(a) non-smoking;
(b) right-handed;
(c) without any signs of malfunction of peripheral blood circulation and functioning of peripheral nervous system;
(d) normal body mass index (BMI value between 18.5 and 24.9);
(e) voluntary agreement for participation in the study;
(f) professional work tasks include at least four hours of computer work daily.

The exclusion criteria were:

(a) health conditions affecting hand peripheral blood circulation (arterial stenosis of any origin, arterial hypertension, hand peripheral mono- and polyneuropathy, cervical radiculopathy, Raynaud syndrome, autoimmune rheumatologic disorders, skin lesions in lower hand and wrist, thyroid hypo- or hyperfunction, diabetes mellitus);
(b) pain in forearm and wrist;
(c) smoking, chewing tobacco, or use of any other drugs;
(d) regular use of pharmaceuticals;
(e) excessive hair growth on lower hand;
(f) overweight or underweight persons (BMI above 25.0 and below 18.4);
(g) fever on the day of testing;
(h) refusal of voluntary participation in the study;
(i) working tasks do not include regular prolonged computer work.

An evaluation of the condition of health was made by a certified physician daily during the duration of the study.

Study participants were instructed not to apply cosmetics to the hands, use any medication, alcohol, or mood-affecting substances, or undergo any physical procedures (like a massage) or major physical activities for at least one day before testing. Heavy meals and stimulating beverages were excluded for at least two hours before measurements. Volunteers were asked to wear comfortable, loose, and sufficiently warm clothes with short sleeves on the day of testing.

### 2.4. Equipment and Procedures

The measurements of skin surface temperature were made by a high-resolution medical digital infrared camera ICI ETI 7320 Pro using specialized imaging analysis software IR Flash Medical Version 2.14.14.4. (Infrared Cameras, Inc.) supplied by manufacturer. The sensitivity of the current camera is 0.027 °C, which allows for the evaluation of minimal thermal changes in skin temperature with high accuracy. The infrared camera was placed on a stable adjustable stand perpendicular to the target. The distance between the camera and the measured skin surface was 135 cm and remained constant throughout the data capture. The evaluated person was seated in an office chair at the table with a polyvinyl chloride laminated surface in a comfortable position at least three meters from the nearest source of heat or cold. There was not any significant source of adverse infrared radiation in the laboratory.

The laboratory environment was under constantly controlled ambient temperature, humidity, and air flow velocity. The measurements of microclimate conditions were made by a Testo 400 precision multi-function measuring device. Mean air temperature during the trials was 23.59 ± 0.38 °C, mean air humidity—32.3 ± 4.1%, and air flow velocity—0.03 ± 0.01 m/s. In parallel, the table surface temperature was followed by infrared imaging (mean value for all trials was 23.21 ± 0.54 °C).

All trials were conducted in the morning, during the first half of the day after the sufficient rest of participants. Each trial lasted for three hours. Thermal images of right forearm and wrist were taken at the beginning of the trial and every 15 minutes over 3 hours. In addition, the temperature of the left hand was checked at the start and at the end of the trial. The trial was repeated with the same person for three days under different ergonomic setups each day. Shortly before the trial they were asked to visit the lavatory. A thermal acclimatization period of 15 minutes was awaited before the trial started.

During the trial participants were asked to surf the Internet for the duration of the trial. They were not assigned with any particular task to avoid additional psychoemotional stress. Participants were asked to mostly use the computer mouse, but were allowed to type with the right hand for short periods to imitate common computer work. Participants were free in the use of their left hand (e.g., for typing on a standard keyboard). 

### 2.5. Ergonomic Scenarios Tested

Measurements were repeated for three days and on each day a different ergonomic computer mouse setup was tested. The position of the right hand was changed every day. Three different scenarios of ergonomic design were tested.

For the first scenario a standard horizontal computer mouse was used without any mouse pad. The right forearm and wrist were placed horizontally on the laminated table, the elbow supported with a height-adjustable chair arm. In such a wrist position, an additional load on the wrist extensor muscles was expected (see Figure S1 in Supplementary Materials). 

For the second scenario a standard horizontal computer mouse with mouse pad and padded wrist support was used. The right forearm and wrist were placed horizontally on the table, the right elbow supported with a height-adjustable chair arm. A reduced load for wrist extensor muscles was expected for this wrist posture, while an adverse compression of blood vessels at the base of the wrist might be assumed (see Figure S2 in Supplementary Materials).

For the third scenario a vertical ergonomic computer mouse without any mouse pad was used. The right forearm and wrist were placed horizontally on the table, the right elbow supported with a height-adjustable chair arm. In such a design, reduced pressure on wrist blood vessels and physiological wrist position without an additional load for extensor muscles was expected (see Figure S3 in Supplementary Materials).

### 2.6. Data Processing and Analysis

**Figure 1:**
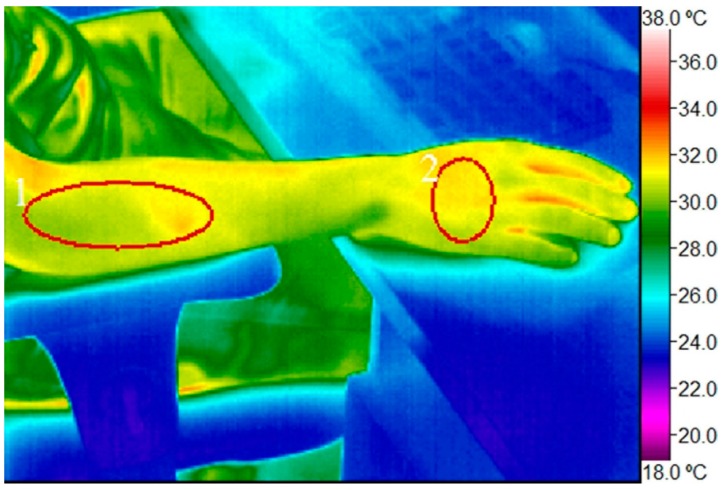
Digital infrared image of the right hand with regions of interest marked (**1**) proximal half of lateral surface of the right forearm (projection site of wrist extensor muscles); (**2**) dorsal surface of the right wrist.

Data on skin surface temperature were acquired from digital infrared images processed with specialized imaging analysis software IR Flash Medical Version 2.14.14.4. The dorsal surface of the right wrist at the level of the metacarpals and the proximal half of the lateral surface of the right forearm (projection site of wrist extensor muscle group) were set as the main regions of interest where the calculations of the skin surface’s mean temperature were made (see Figure 1). Additionally, the point of minimal temperature in the distal parts of the fingers was fixed for more precise evaluation of skin temperature drop in the right wrist; some extra measurements of the mean temperature at the same regions of interest in the left forearm and wrist were also performed. Additional measurements were done at the beginning and end of each trial. Afterwards the data were transposed to Microsoft Excel and the statistical program IBM SPSS Statistics Version 17.0. The significance level was set at 0.05. A mixed multiple-way repeated analysis of variance was performed. Skin surface temperature was evaluated in connection with the time elapsed by correlation analysis. Differences in dynamics of skin surface temperature changes under different ergonomic scenarios were explored by comparative analysis in each person individually, as well as the similarities between persons in each ergonomic scenario being investigated. 

## 3. Results 

Two males (26 and 42 years old; BMI 24.8 and 21.5, respectively) and two females (24 and 26 years old; BMI 23.1 and 20.0, respectively) took part in the current study. Ambient air temperature as well as the temperature of the table did not differ significantly between and during the trials (*p* > 0.05). Air flow velocity was maintained at as low a level as possible (< 0.06 m/s). The deviations in ambient air temperature, humidity, and air flow velocity were considered negligible and did not influence the skin temperature changes. 

**Table 1:** Comparison of mean skin surface temperature in the right and left hands at the regions of interest at the start and 3 hours later by different ergonomic setups (mean ± standard deviation, °C; percentage of temperature changes from starting temperature).

Location and Time of Measurements			Horizontal Computer Mouse without Mouse Pad	Horizontal Computer Mouse and Padded Wrist Support	Vertical Ergonomic Computer Mouse without Mouse Pad
**Right Hand**	Wrist	at the start	31.17 ± 1.42	30.1 ± 1.80	31.97 ± 0.93
		after 3 hours	26.57± 1.84	26.52 ± 1.00	30.06 ± 1.63
		T change	−14.8 %	−11.9 %	−6.0 %
	Forearm	at the start	32.41 ± 0.67	32.15 ± 0.22	32.03 ± 1.29
		after 3 hours	30.56 ± 0.81	30.49 ± 0.65	30.55 ± 1.47
		T change	−5.7 %	−5.2 %	−4.6 %
Left Hand *	Wrist	at the start	31.30 ± 1.75	29.42 ± 2.22	32.13 ± 1.30
		after 3 hours	28.85 ± 2.15	28.85 ± 2.15	31.06 ± 1.16
		T change	−7.8 %	−1.9 %	−3.3 %
	Forearm	at the start	32.11 ± 0.27	31.75 ± 0.38	32.53 ± 0.56
		after 3 hours	31.35 ± 0.70	31.91 ± 0.98	32.22 ± 1.20
		T change	−2.4 %	+0.5 %	−1.0 %

* Simultaneous intermittent tapping on the standard keyboard and free left hand position.

In Table 1 the changes of the skin temperature in the regions of interest are shown, comparing right and left hands by the ergonomic setups. 

**Figure 2:**
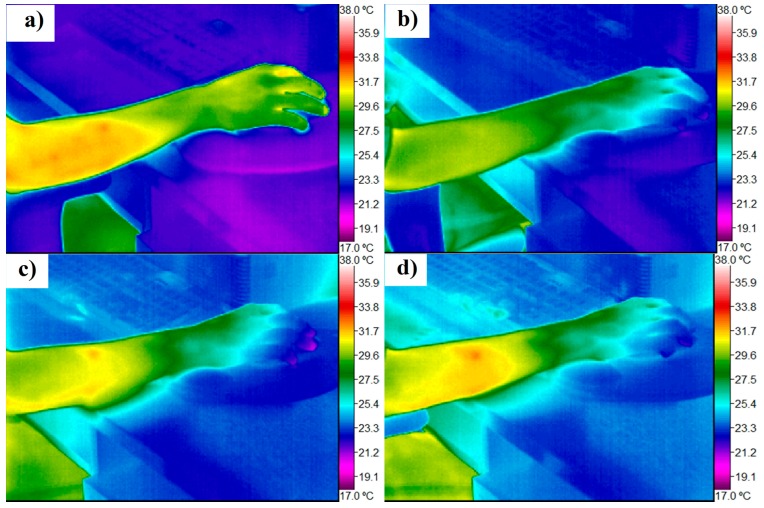
Digital infrared images of the right hand showing the dynamics of skin temperature changes during three hours of work with a horizontal computer mouse and a padded wrist support (**a**) at the start of the trial; (**b**) after 1 hour; (**c**) after 2 hours; and (**d**) after 3 hours.

At the start, the skin temperature of both wrists did not differ significantly among participants and trials (*p* > 0.05). A dramatic decrease in wrist skin temperature was observed after three hours of work at a computer in all cases (*p* < 0.05; see Figure 2). The drop in temperature was not only seen in the right wrist, but also in the left—however, at a lower level (see Table 1). The extent of temperature decrease in the right wrist was different between the ergonomic setups tested. The most prominent decline was observed during work with a horizontal computer mouse with or without a mouse pad (by 14.8% and 11.9%, respectively), while during work with a vertical computer mouse the skin temperature remained more stable (decreasing only by 6.0% in the right wrist). At the same time the decrease of skin temperature in the left wrist was less notable (a maximal decrease of 7.8% observed in the ergonomic design with horizontal computer mouse without mouse pad).

By exploring digital infrared images it became evident that the lowest temperature was reached in the distal parts of the fingers (see Table S1 in Supplementary Materials). A slightly lower starting minimal temperature of fingers was observed in females. A significant drop of minimal temperature was observed in study participants of both genders (achieving even 19.90 °C, which is lower than the ambient temperature of the air in the room and the table). The effect of extremely low temperature in the fingers (below ambient temperature) was observed repeatedly in different persons under ergonomic setups with a horizontal computer mouse, but not with a vertical computer mouse. In many cases, the minimal temperature of fingers in the course of the whole trial was achieved earlier than the three-hour mark, and afterwards temperature slightly increased (for the ranges of mean temperature at the regions of interest see Figures S4–S9 in Supplementary Materials). 

**Figure 3:**
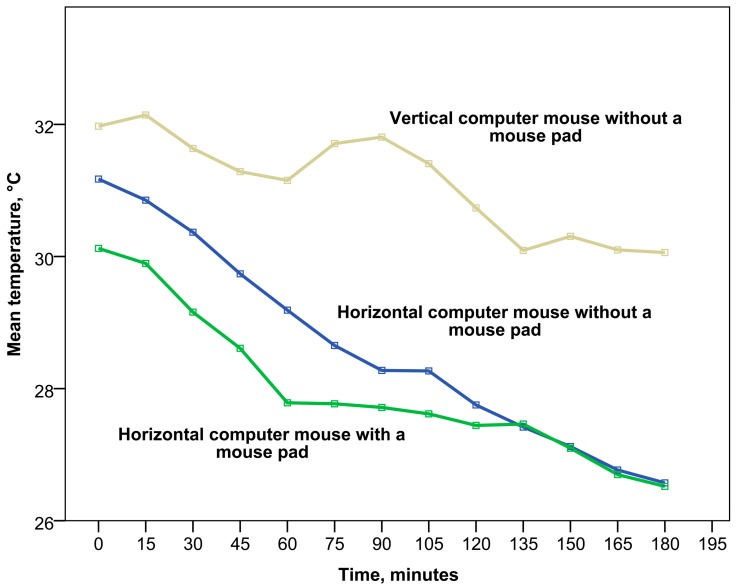
The dynamics of wrist dorsal surface skin temperature changes under different ergonomic setups during work with a computer mouse.

**Figure 4:**
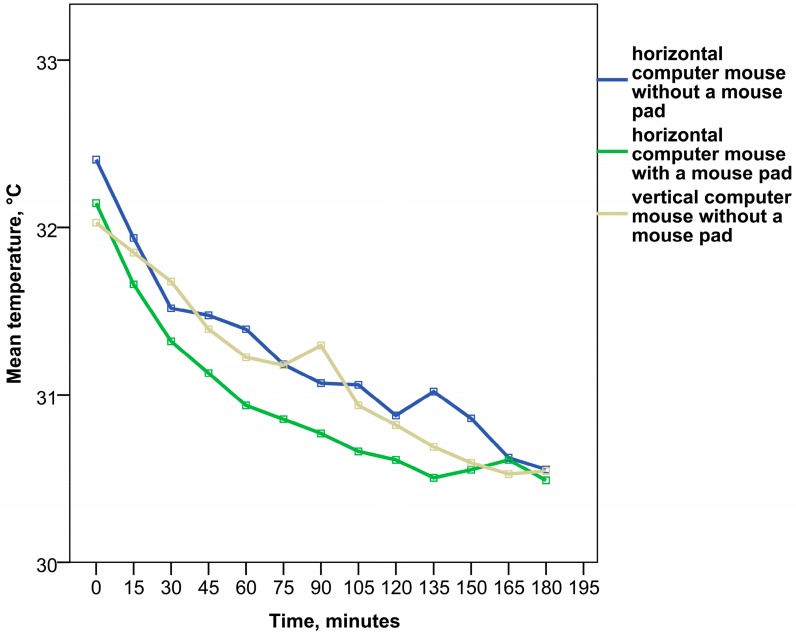
The dynamics of forearm skin temperature changes under different ergonomic setups during work with a computer mouse.

As mentioned previously, the starting skin temperature of the right hand did not differ significantly between participants and trials (*p* > 0.05), but the pattern of temperature decline in time differed significantly according to the ergonomic setup (see Figure 3 and Figure 4). Despite the statistically insignificant differences in starting temperature, an initial increase in wrist skin temperature was observed in most of the trials during the first 15 minutes and afterwards was followed by a sharp decline. The sharpest initial decline was found during the first hour of work with a horizontal computer mouse and padded wrist support. After one hour of work, the temperature became more stable and remained at approximately the same level over one hour. After 135 minutes of work, a new decrease of temperature started and had a similar pattern to working with a horizontal computer mouse without a mouse pad, achieving minimal parameters after three hours. In the case of the horizontal computer mouse with a mouse pad, a moderate negative correlation of wrist temperature with time was observed (Spearman’s correlation coefficient *r_s_* = −0.476, *p* < 0.01). 

Moreover, work with a horizontal computer mouse without a mouse pad was characterized by a stable gradual decrease of wrist temperature (see Figure 3). A statistically significant strong negative correlation of wrist temperature with time was found in this case (Spearman’s correlation coefficient *r_s_* = −0.731, *p* < 0.01). The temperature pattern of the third hour of work with this ergonomic design was identical to work with a horizontal computer mouse and padded wrist support. 

At the same time the analysis of work with a vertical ergonomic computer mouse without a mouse pad showed the highest and most stable temperature of the wrist (see Figure 3). Wrist skin temperature remained quite high during the first hour of work compared to the other ergonomic setups and a slight increase was observed during the second hour. However, a notable decrease of wrist temperature started after 90 minutes of work, reaching its minimum at 135 minutes and then remaining at a low level during the third hour. It is important to stress that even the lowest temperature during the third hour of work with a vertical computer mouse was much higher compared to the ergonomic setups with a horizontal computer mouse (*p* < 0.001). Nevertheless, in the case of work with a vertical ergonomic computer mouse, a moderate negative correlation of wrist temperature with time was also observed (*r_s_* = −0.447, *p* < 0.01).

The analysis of temperature changes in the right forearm revealed a less evident decrease of temperature compared to the distal portions of the same hand. The skin temperature of the dorsal surface of the right forearm was statistically significantly higher than in the dorsal surface of the wrist in trials with a horizontal computer mouse (*p* < 0.001), while in trials with a vertical computer mouse such differences were not observed (*p* > 0.05) and the temperature at both locations was very similar (*p* < 0.001). Nevertheless, some differences in the dynamics of temperature changes between ergonomic designs were observed as well (see Figure 4 and Figures S7–9 in Supplementary Materials). A continuous, gradual decrease of forearm skin temperature was found from the very beginning of work with a horizontal computer mouse and padded wrist support. A slight increase of temperature started only after 135 minutes of work and lasted for half an hour, followed by temperature decrease. In this ergonomic design a strong statistically significant correlation of forearm skin temperature with time was observed (Spearman’s correlation coefficient was found to be *r_s_* = −0.625, *p* < 0.01).

In the case of work with a horizontal computer mouse without a mouse pad, forearm temperature decreased less gradually with short periods of increase and in general was higher than in work with padded wrist support (*p* < 0.05). A strong statistically significant correlation of forearm skin temperature with time was observed as well (Spearman’s correlation coefficient was *r_s_* = −0.632, *p* < 0.01).

The work with a vertical ergonomic computer mouse without a mouse pad revealed a temperature pattern of the forearm similar to work with a horizontal computer mouse without mouse pad and only a weak statistically significant correlation of temperature with time was found in this case (*r_s_* = −0.330, *p* < 0.05). In general, forearm temperature was higher than with work with a horizontal computer mouse with padded wrist support (*p* < 0.05), but did not differ significantly from work with a horizontal computer mouse without a mouse pad (*p* > 0.05). After three hours of work the temperature of the forearm was lower, as at the start of the trial (*p* < 0.05), and did not differ among ergonomic setups (*p* > 0.05).

## 4. Discussion

The study revealed a notable decrease of hand skin surface temperature during prolonged work with a computer mouse. Human skin temperature is a very labile parameter and may be influenced by many factors [48]. Therefore, it is important to discuss possible reasons for temperature decline while evaluating the thermal effect of prolonged computer mouse work. The most prominent agents involved in skin temperature changes might be [48]:

(1) Environmental factors (ambient air temperature, air humidity, air flow velocity, room size, sources of radiation, *etc*.);

(2) Individual factors: (a) intrinsic (general—sex, age, medical history, inflammatory conditions and fever, hormonal status, metabolic rate, emotions, circadian rhythm, *etc*.; local—sickness of adipose tissue layer, condition of muscles and their volume, blood supply and innervation with local vegetative reactions in skin vessels, local inflammatory processes, hair density, *etc*.); (b) extrinsic (intake factors, therapy, physical activity, *etc*.).

(3) Technical factors.

Our study was designed to investigate the impact of ergonomic factors on forearm and wrist temperature by controlling for other possible influencing conditions. The trials were conducted in laboratory settings. In order to minimize the effect of technical and environmental factors, the study was organized taking into account the recommendations for medical thermography implementation [49,50,51]. Environmental factors were controlled, maintaining stable air temperature, air humidity, and air flow velocity throughout the course of the trials. Minor variations in these parameters were considered to be insignificant and did not influence the measurements. The air temperature was kept within the comfortable limits for sedentary work recommended by most of the authorities [35,36,37,38]. Despite controlling for environmental and technical factors, a significant decrease of hand skin temperature was observed.

The regions of interest were chosen to avoid the influence of large superficial blood vessels on skin temperature while targeting and evaluating the muscular load of the forearm. Fingers were not chosen as a specific region of interest due to there being too high a variability of skin temperature between persons and trials. During the measurements, regions of interest were drawn with caution in order not to leave the outlines of the body on the infrared images where temperature parameters could be lower. 

Individual factors of study participants could be of greater importance for the interpretation of study results. To avoid the impact of side effects from vegetative reactions (e.g., after meal or hot beverage consumption, as well as the need for emptying the bladder or bowels), study participants were instructed not to eat or drink before the trial and to visit the lavatory shortly before the start of the trials. All study participants were within a normal range of body mass index and had musculature developed to a similar degree. The hair density in the region of interest was similar for all persons. Careful medical examination before each trial excluded other medical conditions that might influence the results. At the same time the unnecessary compression and rubbing of tissues was avoided during the examination and preparation for the study. The effect of gender was evaluated by including two males and two females in the study (for the range of individual variability see Table S1 and Figures S4–S9 in Supplementary Materials). Some individual features of adaptive reactions could be observed during the current study, but similar clinically important patterns were recognized. One study participant was slightly older than the others (42 years *vs.* 24 and 26 years) but careful medical examination did not reveal any differences in the skin condition or blood supply that might influence the results. Moreover, the pattern of temperature changes of the older participant was very similar to the pattern of younger ones. A slightly lower starting temperature of the wrist was observed in females, which might be assumed as being due to a more fine structure and lower circulating volume of warm blood in the wrist tissues of examined females than in males. Hormonal differences might influence the skin temperature as well. Otherwise the tendency in thermal reaction on prolonged work with a computer mouse was similar for both genders. One of the most influential confounding factors might be the circadian rhythms of study participants [48]. In studies from previous years it has been shown that skin temperature in the distal regions of the body gradually decreases from approximately 8 a.m. until 6 p.m. [48,52,53]. Our trials were conducted in the morning, during the first half of the day, therefore the observed decrease of skin temperature might be partly caused by circadian rhythms. Nevertheless, the skin temperature reported in previous research [48] was not as low as in our trials. 

Taking into account the small number of study participants, we can only assume the actual mechanisms of and reasons for the temperature decrease. Our study findings suggest that the effect of sedentary behavior and motionless work with a computer mouse might be the cause of hypothermia of the hand, because sedentary behavior provides less active energy metabolism and lower heat production. The main reason for temperature decline in distal parts of the hand might be impaired blood circulation in the wrist due to additional compression of blood vessels caused by the wrist position. This was confirmed by a more rapid skin temperature decline when working with a horizontal computer mouse and a padded wrist support. Work with a horizontal computer mouse requires an internal rotation of the wrist in such a way that increases pressure in the soft tissues of the wrist and forearm. At the same time, work with a vertical computer mouse keeps the wrist in an anatomically neutral position without additional pressure and rotation. In this way, during trials with a vertical computer mouse, the wrist temperature remained higher for a longer period. However, despite the ergonomic design, wrist temperature declined dramatically after two hours of motionless work and reached an extremely low level (close to ambient temperature). This observation might indicate the need for active movement for sustaining appropriate blood supply for peripheral body parts during sedentary work already after approximately one hour of continuous work at a computer. Our results are partly in agreement with the study of Huizenga *et al.* (2004), where skin temperature was tested under different thermal conditions for a prolonged time [54].

Hypothermia was greater in the wrist than in the forearm and might be explained by a better blood supply in the forearm while it is the more proximal body part [48]. At the same time, minor differences in forearm temperature between ergonomic designs were observed too. A higher temperature was observed when working with a vertical computer mouse (probably because of a warmer hand in general or the unusual position of the hand for study participants, which put a greater load on forearm muscles) and in work with a horizontal computer mouse without a mouse pad. Work with a horizontal computer mouse without a mouse pad puts a more intensive load on extensor muscles for finger extension during computer mouse movements and for targeting the mouse buttons, which might result in higher skin temperature above loaded muscles. Conversely, during work with a horizontal mouse combined with a padded wrist support the position of the lower arm is more linear, which requires less effort for extensor muscles in the forearm. Subsequently, we observed lower temperatures in the forearm.

The lowest temperature of the wrist was observed in the distal parts of the fingers. Moreover, the temperature of fingers became extremely low (close to the ambient temperature and even slightly lower) after prolonged work at a computer. We suppose that the probable explanation for the extremely low temperature of the fingers during prolonged computer work might be the spastic reactions of peripheral blood vessels due to static wrist position for too long a period of time, as well as the activation of the sympathetic nervous system and, as a result, an extremely impaired blood flow. The reasons for such phenomena should be explored and discussed in further investigations.

Our observations indicated a decrease of skin temperature not only in the right hand, but in the left hand too. This might be explained by simultaneous vasomotor reactions of both hands. These findings coincide with the Isii *et al.* (2007) study of hand temperature changes in contralateral hand after ice-water hand immersion [55]. Partly sedentary behavior might contribute to a cooling of the whole body [56].

There were a limited number of study participants in our pilot observational study. We investigated skin temperature changes in four people only, but despite the small number of study participants our trials revealed prominent, statistically significant differences and a quite well repeatable pattern of temperature dynamics with moderate individual variability. Prolonged low temperature and probably impaired blood supply to peripheral tissues in hands may be important contributing factors for the development of tissue damage, e.g., small nerve fibers. A normal body temperature is needed for optimal action of the biochemical reactions in a cell [31,32]. Taking into account the quite high prevalence of work-related carpal tunnel syndrome and musculoskeletal disorders of the hand in computer users [1,22], our findings might be clinically important for further research on the methods for prevention of work-related disorders. The mechanisms of this process should be investigated in detail and might be highly important for occupational health settings. Larger studies should be conducted to further investigate the thermal effects of prolonged computer mouse work, including a broader age groups, other models of ergonomic setups, and simultaneously investigating the blood circulation in hands. 

## 5. Conclusions 

Our study findings have shown that the skin temperature of the dorsal surface of the dominant hand (measured at wrist level and proximal half of the forearm) was strongly negatively correlated with time spent working with a computer mouse. Nevertheless, irrespective of the ergonomic setups tested, the skin temperature of the hand markedly decreased after one hour of continuous sedentary work, indicating the need for breaks with active rest. The work with a vertical ergonomic computer mouse preserved a more stable and higher temperature of the wrist, while a duration of continuous work with a computer mouse of more than two hours caused an extremely low temperature in the distal parts of the hand, especially while working with a horizontal computer mouse, despite the comfortable ambient air temperature. The employment of a mouse pad with a padded wrist support might cause additional compression of the wrist base blood vessels, resulting in a sharp decline of wrist temperature during the first hour of work. Further investigations on hand temperature changes under different ergonomic setups and controlled environmental conditions are needed to verify these preliminary findings.

## Supplementary Files

Supplementary File 1
