# Supplementary material for: Wrist Hypothermia Related to Continuous Work with a Computer Mouse: A Digital Infrared Imaging Pilot Study
Source: Int J Environ Res Public Health. 2015 Aug 7;12(8):9265–81. doi: 10.3390/ijerph120809265 (PMC4555279; doi:10.3390/ijerph120809265)
Supplement: Supplementary File 1 [file ijerph-12-09265-s001.pdf]

Supplementary Information

Wrist Hypothermia Related to Continuous Work with a Computer Mouse: A Digital Infrared Imaging Pilot Study

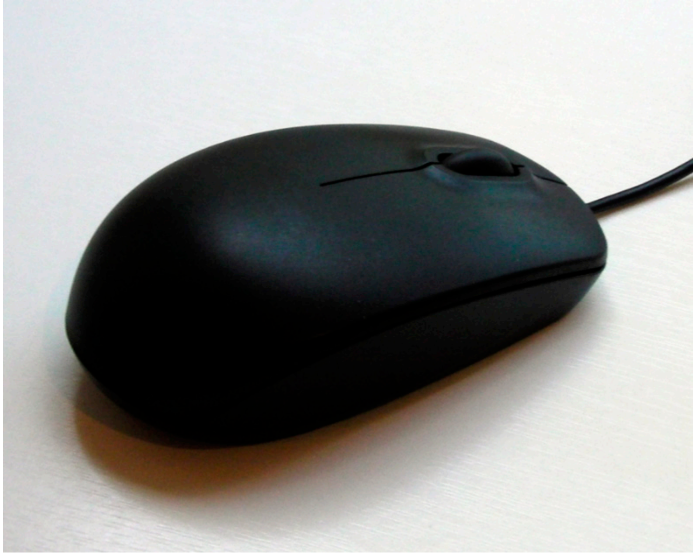

Figure S1. A horizontal computer mouse without a mouse pad (1st ergonomic scenario).

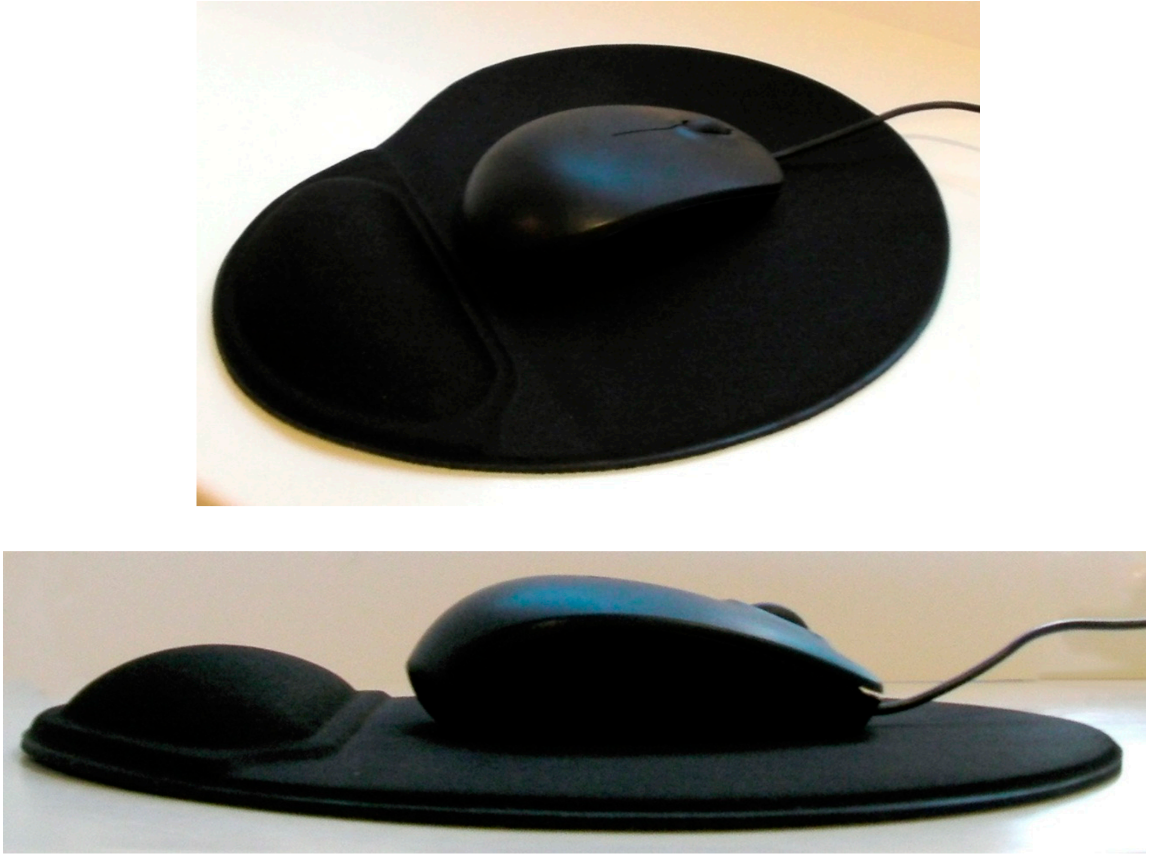

Figure S2. A horizontal computer mouse with a mouse pad and padded wrist support (2nd ergonomic scenario).

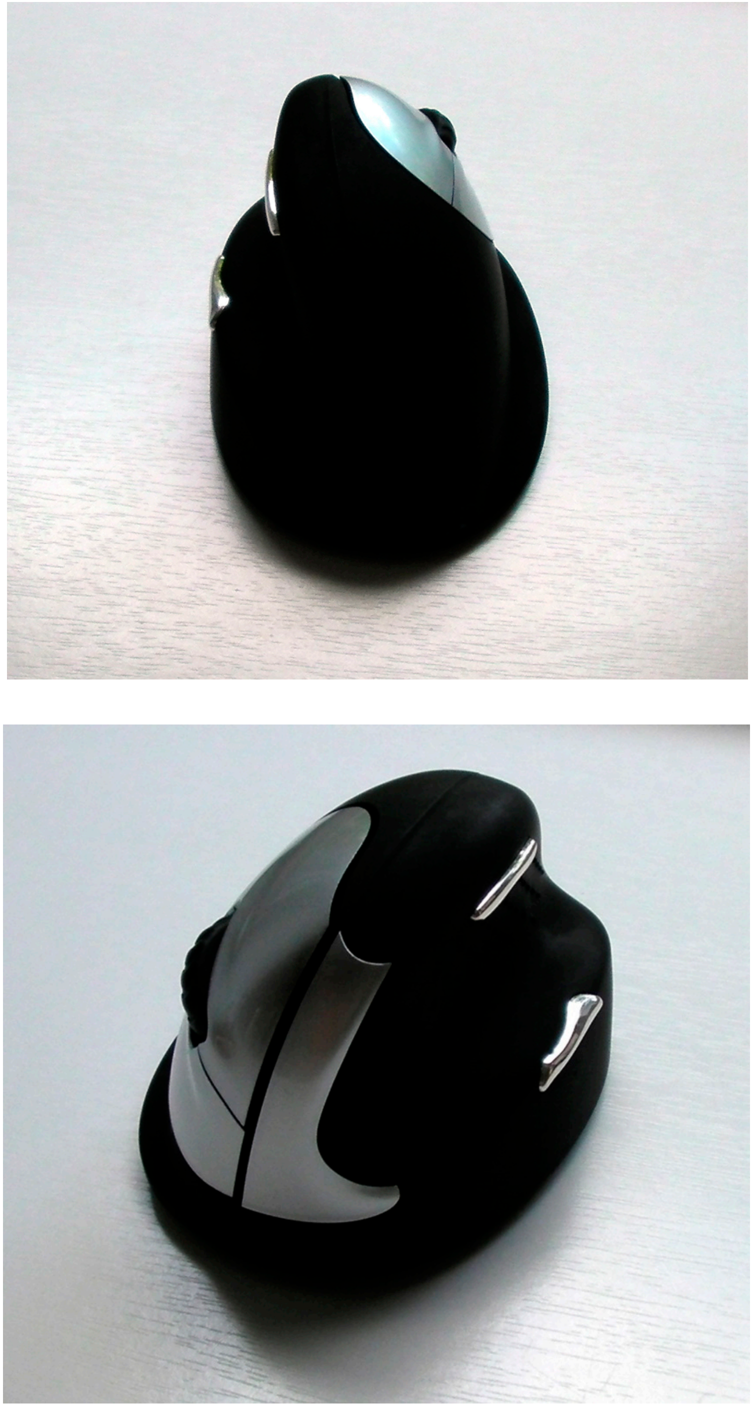

Figure S3. A vertical ergonomic computer mouse without a mouse pad (3rd ergonomic scenario).

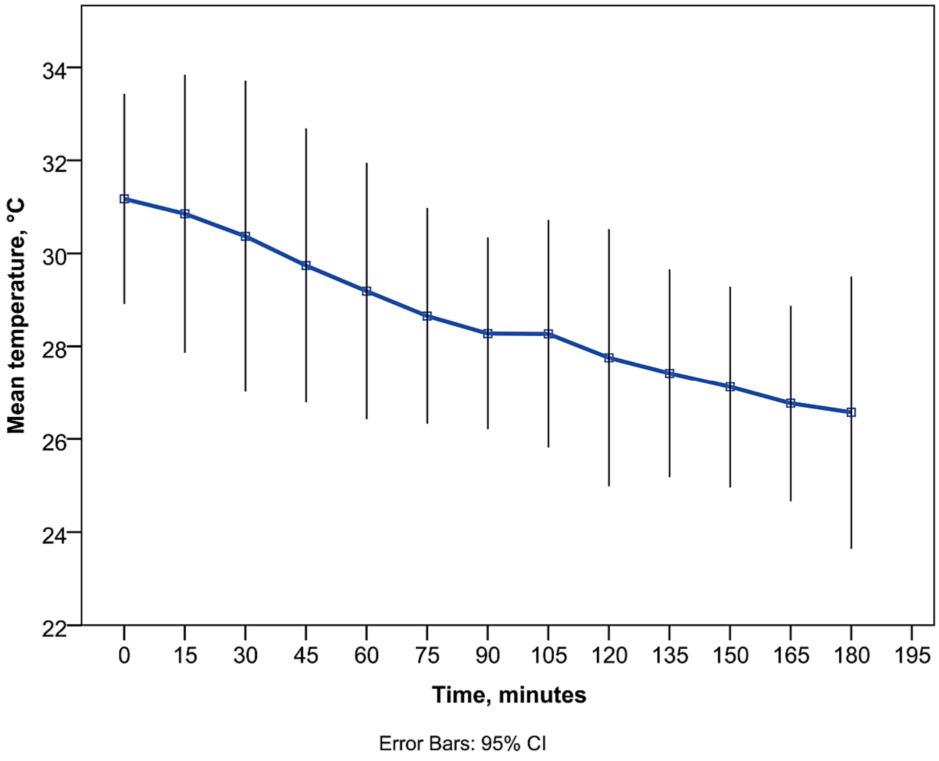

Figure S4. The dynamics of skin temperature changes of the wrist dorsal surface during work with a horizontal computer mouse without a mouse pad (mean temperature in the region of interest and 95% confidence intervals (CI) are shown by error bars).

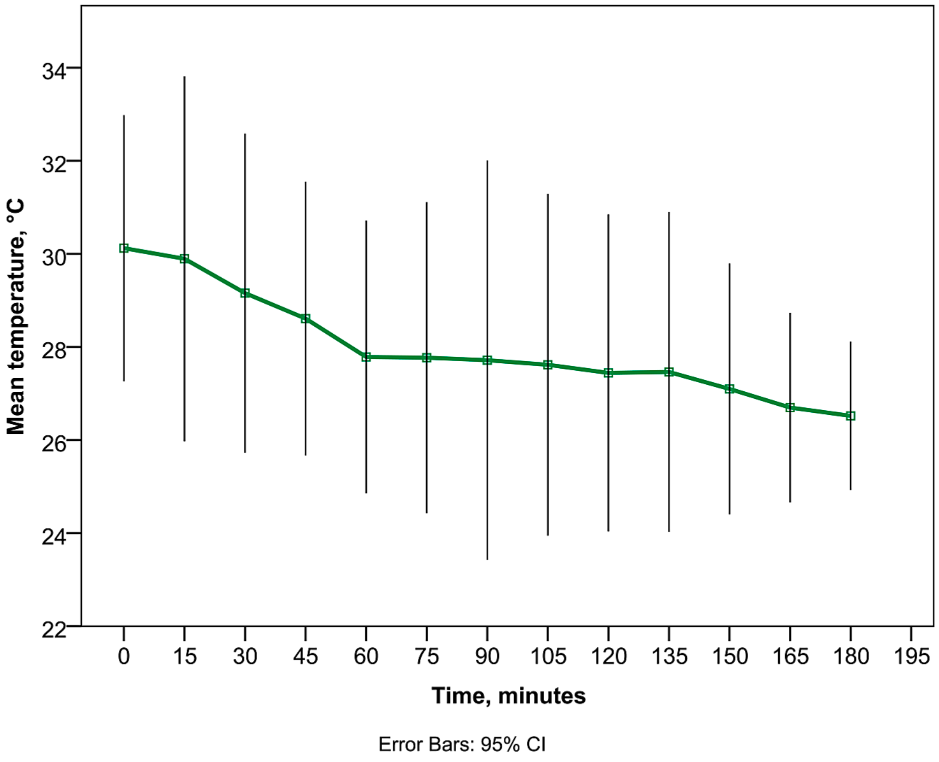

Figure S5. The dynamics of skin temperature changes of the wrist dorsal surface during work with a horizontal computer mouse with a mouse pad and padded wrist support (mean temperature in the region of interest and 95% confidence intervals (CI) are shown by error bars).

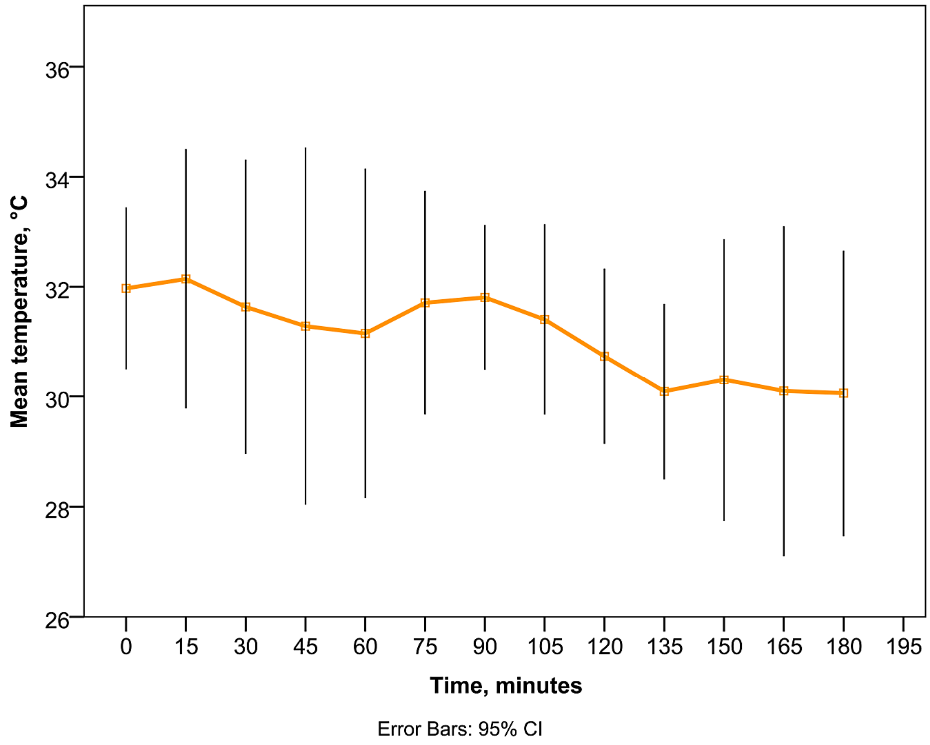

Figure S6. The dynamics of skin temperature changes of the wrist dorsal surface during work with a vertical ergonomic computer mouse without a mouse pad (mean temperature in the region of interest and 95% confidence intervals (CI) are shown by error bars).

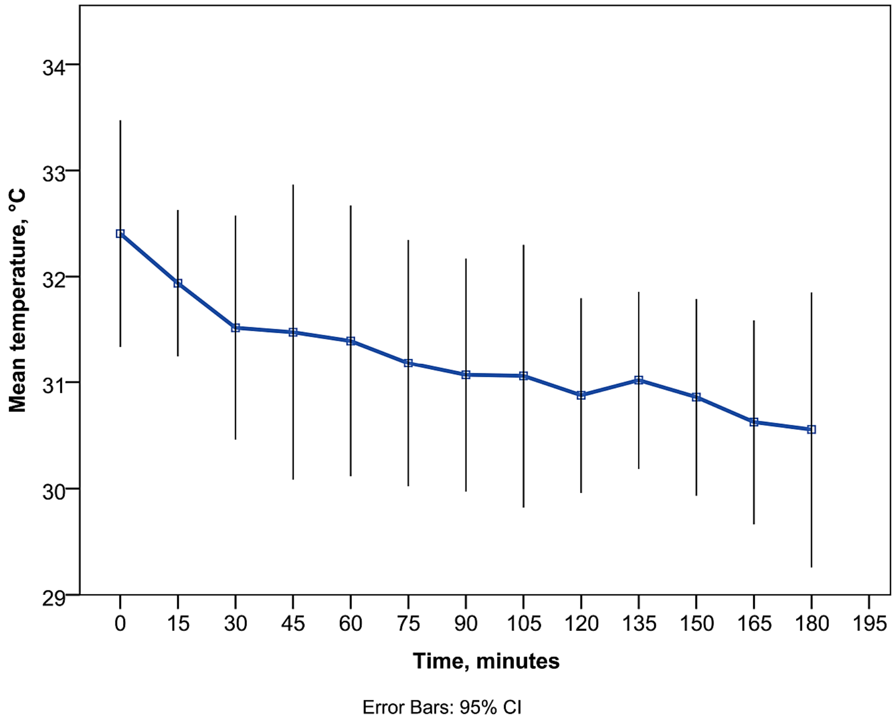

Figure S7. The dynamics of skin temperature changes in the lateral surface of the proximal half of the right forearm during work with a horizontal computer mouse without a mouse pad (mean temperature in the region of interest and 95% confidence intervals (CI) are shown by error bars).

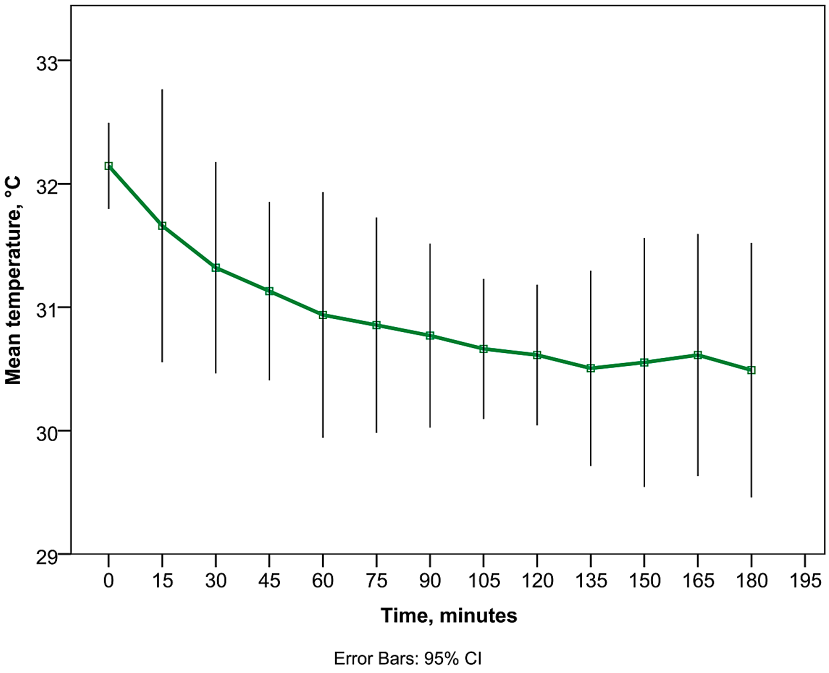

Figure S8. The dynamics of skin temperature changes in the lateral surface of the proximal half of the right forearm during work with a horizontal computer mouse with a mouse pad and padded wrist support (mean temperature in the region of interest and 95% confidence intervals (CI) are shown by error bars).

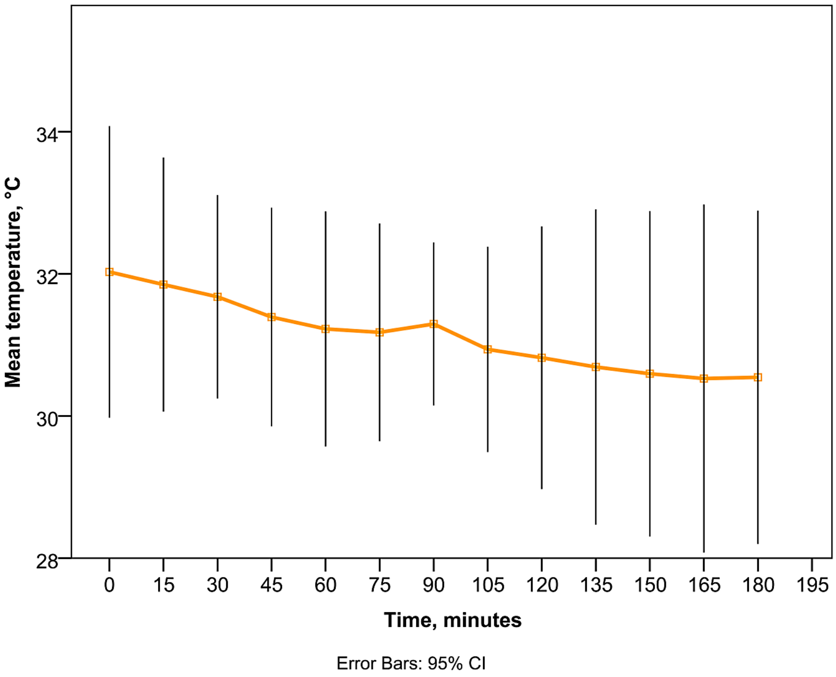

Figure S9. The dynamics of skin temperature changes in the lateral surface of the proximal half of the right forearm during work with a vertical ergonomic computer mouse without a mouse pad (mean temperature in the region of interest and 95% confidence intervals (CI) are shown by error bars).

Table S1. Skin surface minimal temperature in the distal portions of the right fingers for each study participant at the start and after three hours, divided by different ergonomic setups and gender (actual temperature (°C) and percentage of temperature decrease from the start of work are shown).

**Ergonomic Scenario**	**Right Hand**					
	**Males**			**Females**		
	**At the Start**	**After 3 Hours**	**%**	**At the Start**	**After 3 Hours**	**%**
horizontal computer mouse without mouse	32.15	24.13	–25.7%	28.30	22.40	–20.8%
pad	32.67	20.03	–38.7%	28.98	19.90	–31.3%
horizontal computer mouse with mouse pad	31.30	19.60	–37.4%	23.50	23.60	+0.4%
and padded wrist support	26.68	23.57	–11.7%	29.32	20.65	–29.6%
vertical ergonomic computer mouse without	33.55	23.14	–31.0%	28.40	23.32	–17.9%
mouse pad	31.40	31.30	–0.3%	31.62	26.49	–16.2%

© 2015 by the authors; licensee MDPI, Basel, Switzerland. This article is an open access article distributed under the terms and conditions of the Creative Commons Attribution license (http://creativecommons.org/licenses/by/4.0/).
